# Isolation of a Complex Formed Between *Acinetobacter baumannii* HemA and HemL, Key Enzymes of Tetrapyrroles Biosynthesis

**DOI:** 10.3389/fmolb.2019.00006

**Published:** 2019-02-26

**Authors:** Caterina Nardella, Dalila Boi, Martino L. di Salvo, Anna Barile, Jörg Stetefeld, Angela Tramonti, Roberto Contestabile

**Affiliations:** ^1^Laboratory Affiliated to Istituto Pasteur Italia-Fondazione Cenci Bolognetti, Dipartimento di Scienze Biochimiche “A. Rossi Fanelli”, Sapienza Università di Roma, Rome, Italy; ^2^Department of Chemistry, University of Manitoba, Winnipeg, MB, Canada; ^3^Istituto di Biologia e Patologia Molecolari, Consiglio Nazionale delle Ricerche, Rome, Italy

**Keywords:** *Acinetobacter baumannii*, pyridoxal 5′-phosphate-dependent enzymes, HemA, HemL, glutamyl-tRNA reductase, glutamate-1-semialdehyde aminomutase, protein complex

## Abstract

Plants, algae and most bacteria synthesize 5-aminolevulinic acid (ALA), the universal precursor of tetrapyrroles such as heme, chlorophyll and coenzyme B_12_, by a two-step transformation involving the NADPH-dependent glutamyl-tRNA reductase (HemA), which reduces tRNA-bound glutamate to glutamate-1-semialdehyde (GSA), and the pyridoxamine 5′-phosphate-dependent glutamate-1-semialdehyde-2,1-aminomutase (HemL), responsible for the isomerization of GSA into ALA. Since GSA is a very unstable compound at pH values around neutrality, the formation of a HemA-HemL complex has been proposed to occur, allowing for direct channeling of this intermediate from HemA to HemL. Experimental evidence of the formation of this complex has been obtained with the enzymes from *Escherichia coli* and *Chlamydomonas reinhardtii*. However, its isolation has never been attained, probably because HemA is degraded when intracellular heme accumulates. In this work, we devised a co-expression and co-purification strategy of HemA and HemL from *Acinetobacter baumannii*, which allowed the isolation of the HemA-HemL complex. Our results indicate that HemA is stabilized when co-expressed with HemL. The addition of citrate throughout the expression and purification procedure further promotes the formation of the HemA-HemL complex, which can be isolated in fair amount for functional and structural studies. This work lays the bases for a rational design of HemA-HemA inhibitors to be developed as antibacterial agents against *A. baumannii*, a multidrug resistant opportunistic pathogen responsible for a broad range of severe nosocomial infections.

## Introduction

5-Aminolevulinic acid (ALA) is the universal precursor in the tetrapyrrole biosynthesis pathway. In animals, fungi and few bacteria, ALA is synthesized in the one-step condensation of succinyl-CoA and glycine catalyzed by ALA synthase (Radin et al., 1950). On the other hand, plants, algae, and most bacteria synthesize ALA by a two-step transformation, starting from tRNA-bound glutamate. In these organisms, glutamyl-tRNA is reduced to glutamate-1-semialdehyde (GSA) by the NADPH-dependent glutamyl-tRNA reductase (HemA) (Jahn et al., 1992). Then, GSA is isomerized to ALA by the pyridoxamine 5′-phosphate-dependent glutamate-1-semialdehyde-2,1-aminomutase (HemL) (Kannangara et al., 1988; Grimm et al., 1989). At physiological pH, GSA is a very unstable amino aldehyde that easily degrades generating toxic products within the cells (Pugh et al., 1991). This is a typical case in which substrate channeling is called in. In fact, the formation of a complex between HemA and HemL would allow a direct transfer of GSA from the active site of HemA to HemL, without the need for this unstable metabolite to diffuse through the medium. Computer-aided modeling, based on X-ray crystallographic structures of HemA from the hyperthermophilic archaeon *Methanopyrus kandleri* (Moser et al., 2001) and HemL from the cyanobacterium *Synechococcus* sp. 6301 (Hennig et al., 1997), has been used to predict that the two enzymes could interact to accomplish the channeling of GSA (Moser et al., 2001). The homodimeric HemA has an unusual overall V-shape. Each monomer is a leg of the V and consists of three domains linked by a long “spinal” α-helix. The N-terminal catalytic domain specifically binds the glutamate moiety of the substrate and contains the catalytic cysteine responsible for the attack to the aminoacyl linkage of the natural substrate glutamyl-tRNA. The resulting thioester intermediate is ultimately reduced by direct hydride transfer from NADPH, supplied by the NADPH binding domain to form the product GSA. The third domain of *M. kandleri* HemA is responsible for dimerization through three α-helices from two neighboring monomers (Moser et al., 2001, 2002). The open V-shape of HemA provides a complementary surface for the association of a HemL homodimer. HemL is a member of fold-type I vitamin B_6_-dependent enzymes (Grishin et al., 1995). It catalyzes the isomerization of GSA to ALA via a reaction closely analogous to pyridoxal 5′-phosphate (PLP)-dependent transamination. However, unlike aminotransferases, HemL catalyzes an intramolecular exchange of the amino and carbonyl moieties which are both present in the substrate. In particular, the catalytic cycle starts with GSA bound to the pyridoxamine 5′-phosphate (PMP) form of the enzyme, and goes through the formation of the 4,5-diaminovalerate (DAVA) intermediate and a transitory PLP-form of the enzyme (Pugh et al., 1992; Tyacke et al., 1993). The HemL dimer shows an interesting structural asymmetry that involves an active site loop, whose function is to prevent DAVA dissociation from the active site (Hennig et al., 1997; Contestabile et al., 2000a). This asymmetry is related to the form of the cofactor present at the active site (PLP or PMP), and therefore to the phases of the catalytic cycle (Stetefeld et al., 2006; Sorensen and Stetefeld, 2011; Campanini et al., 2013). The volume occupied by the HemL dimer is compatible with the open space delimited by the two legs of the V-shaped HemA (Moser et al., 2001). Moreover, the location of the active site entrance of HemL in the HemA-HemL complex model is positioned opposite to a partly open depression of HemA catalytic domain, suggesting that the produced GSA may leave HemA from a “back door” of domain I and be directly channeled to HemL (Moser et al., 2001). The formation of the complex was experimentally demonstrated with *Escherichia coli* HemA and HemL (Lüer et al., 2005) and with the two enzymes from the unicellular alga *Chlamydomonas reinhardtii* (Nogaj and Beale, 2005) by co-immunoprecipitation experiments, gel permeation chromatography and sucrose gradient ultracentrifugation. Nevertheless, the HemA-HemL complex has never been isolated nor characterized.

HemA and HemL are not found in humans but are essential for tetrapyrroles biosynthesis in most bacteria and plants, in which formation of ALA is considered to be the rate-limiting step of heme and chlorophyll biosynthesis. Therefore, their selective inhibition represents an attractive target for development of a new generation of antibiotics and herbicides (Gough et al., 1993). However, to date, little effort has been devoted to this task and no efficient and specific *in vitro* inhibitors of the single HemA or HemL enzymes have been found (Gardner et al., 1988; Hoober et al., 1988; Loida et al., 1999; Contestabile et al., 2000b). Given the strong possibility that HemA and HemL exist as a complex inside the cells, whose structure and properties may be considerably different from those of the single enzymes, a rational approach to the development of inhibitors requires a detailed characterization of the HemA-HemL complex. The present work has the intent to develop a strategy to isolate the complex formed by HemA and HemL of *Acinetobacter baumannii*. This is an important Gram-negative opportunistic pathogen causing a broad range of severe nosocomial infections, including skin and soft tissue infections, wound infections, urinary tract infections, secondary meningitis, ventilator associated pneumonia and bloodstream infections. Moreover, *A. baumannii* is classified by the Infectious Diseases Society of America as one of the six most important multidrug resistant microorganisms in hospitals worldwide (Antunes et al., 2014).

## Materials and Methods

### Materials

All chemicals and ingredients for bacterial growth were from Sigma-Aldrich, except tRNA from *Saccharomyces cerevisiae*, which was from Applied Biosystems. HisTrap affinity columns for purification of 6xHis-tagged protein were from GE Healthcare. Oligonucleotides (Table 1) and DNA sequencing were from Eurofins MWG Operon (Ebersberg, Germany). The L-4-aminohex-5-enoate from which L-glutamate-1-semialdehyde (GSA) was prepared by ozonolysis (Pugh et al., 1991) was a gift from Hoechst Marion Roussel (Cincinnati, OH).

**Table 1:** Plasmids and oligonucleotides used in this work.

**PLASMIDS USED**	
pET28b(+)	Expression vector.
pET28*hemL*_6xHis_	pET28 containing the coding sequence of the *hemL* gene from *A. baumannii* ligated to the *Nde*I and *BamH*I restriction sites.
pET28*hemA*_6xHis_	pET28 containing the coding sequence of the *hemA* gene from *A. baumannii* ligated to *Nde*I and *BamH*I restriction sites.
pET28*hemL*_6xHis_-*hemA*_6xHis_	pET28 containing the coding sequence of *hemL* and *hemA* genes from *A. baumannii*. Both genes carry the 6xHis encoding sequence.
pET28*hemL-hemA*_6xHis_	pET28 containing the coding sequence of *hemL* and *hemA* genes from *A. baumannii*. Only *hemA* carries the 6xHis encoding sequence.
pET43*hemA*_NusA_	pET43 containing the coding sequence of the *hemA* gene from *A. baumannii* ligated to *Sma*I and *BamH*I restriction sites.
**OLIGONUCLEOTIDES USED**	
*AbhemA*_for	ggcatATGTCTTTCTTTGCATTGGGTG
*AbhemA*_rev	ggggatccTTAACGTTTGGGTTTTCGCTC
*AbhemL*_for	ggcatATGAGTTTATCTCCAAAGCAAG
*AbhemL*_rev	ggggatccTTACTTCATTTCGGCGAAGG
*AbhemA*_for2	GGGAATTCAAGAAGGAGATATACCATGGGC
*AbhemA*_rev2	ggctcgagTTAACGTTTGGGTTTTCGCTC
*AbhemL*_NOTAG__for	aagaaggagatataccATGAGTTTATCTCCAAAGCAAG
*AbhemL*_NOTAG__rev	TGGAGATAAACTCATggtatatctccttcttaaagttaaac
*AbhemA*pET43_for	ggggcTCTTTCTTTGCATTGGGTGTC

### Cloning of HemA and HemL From *Acinetobacter baumannii*

**Scheme 1:**
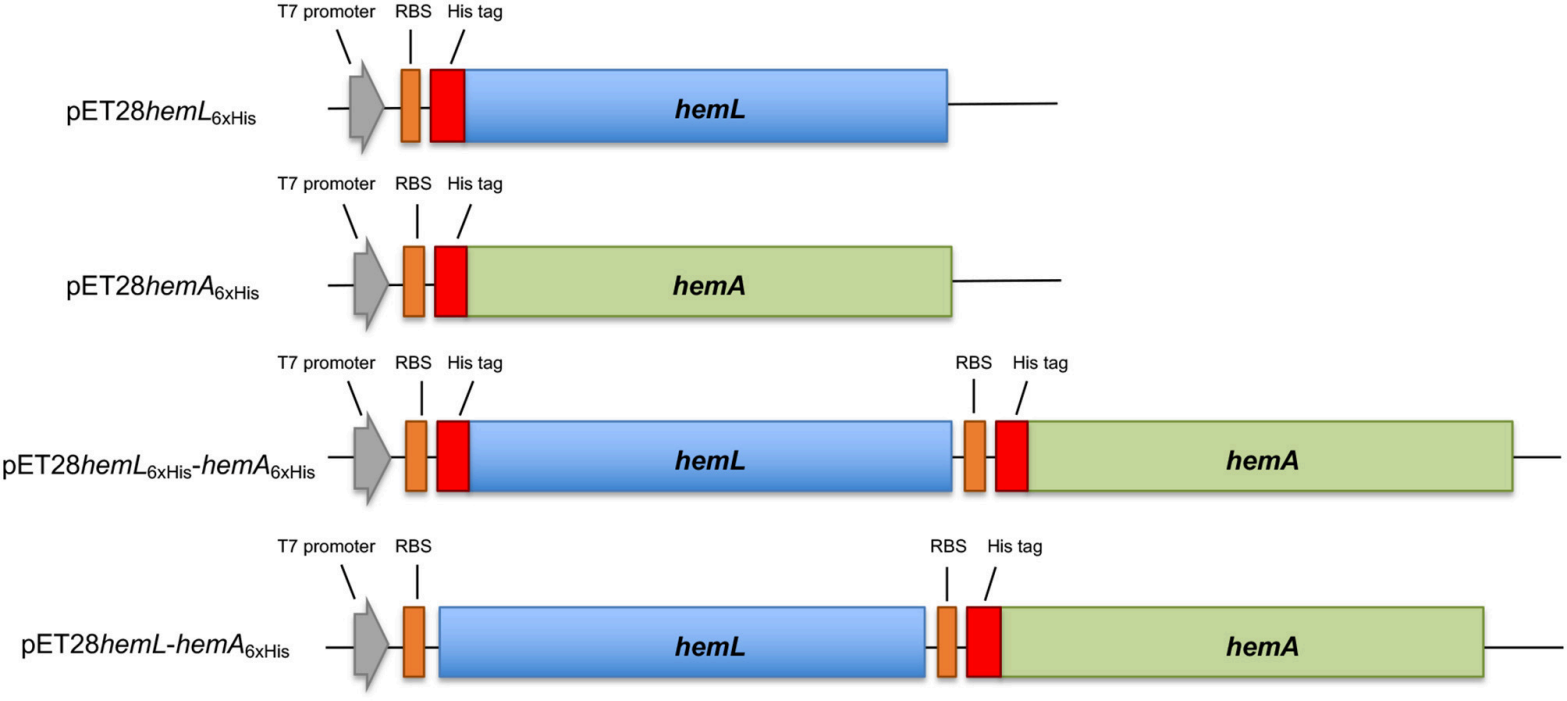
Schematic representation of constructs used in this work. The *hemL* (in blue) and *hemA* (in green) genes were cloned in the pET28 plasmid, containing the T7 promoter (gray arrow) and ribosomal binding site (orange box) with or without the six histidine tag-coding sequence (red box).

The genomic *A. baumannii* DNA was purified following the procedure indicated for *Escherichia coli*. The coding sequences of the *hemA* and *hemL* genes from *A. baumannii* (strain ATCC 19606) were amplified by PCR using primers *AbhemA*_for/*AbhemA*_rev and *AbhemL*_for/*AbhemL*_rev (Table 1). Amplicons were inserted into a pET28b(+) vector between *Nde*I and *BamH*I restriction sites to obtain plasmids pET28*hemA*_6xHis_ and pET28*hemL*_6xHis_ (Scheme 1).

Plasmid pET28*hemL*_6xHis_-*hemA*_6xHis_ (Scheme 1) was obtained by inserting in pET28*hemL*_6xHis_, downstream of the *hemL* stop codon between *Eco*RI and *Xho*I restriction sites, the fragment amplified from pET28*hemA*_6xHis_ (using primers *AbhemA*_for2/*AbhemA*_rev2) containing the ribosomal binding site, the His tag sequence and the *hemA* gene. Plasmid pET28*hemL-hemA*_6xHis_ (Scheme 1) was obtained by deletion mutagenesis from pET28*hemL*_6xHis_-*hemA*_6xHis_ using In-Fusion technology (Takara) and oligonucleotides *AbhemL*_NOTAG__for/*AbhemL*_NOTAG__rev (Table 1). This allows the expression of *Ab*HemL with any tag. The pET43*hemA*_NusA_ plasmid was obtained by inserting the fragment amplified from pET28*hemA*_6xHis_ using primers *AbhemA*pET43_for and *AbhemA*_rev into the pET43 plasmid, between *Sma*I and *BamH*I restriction sites. The nucleotide sequence of all the inserts was determined for both strands. *E. coli* BL21(DE3) competent cells were used as recipient cells for protein expression.

### Purification of AbHemA and AbHemL

An overnight culture (40 mL) of *E. coli* BL21(DE3) cells transformed with appropriate plasmids was used to inoculate 4 L of Luria–Bertani broth containing kanamycin (40 mg·L^−1^). Bacteria were allowed to grow for ~3 h at 37°C (until their OD_600_ reached to ~0.6), then the growing temperature was lowered to 28°C and the expression of HemA/L induced with 0.2 mM isopropyl thio-β-D-galactoside (IPTG). Bacteria were harvested after 18 h and suspended in 50 mM Na-phosphate buffer at pH 8.0 containing 300 mM NaCl (buffer A) and Complete protease inhibitor (one cocktail tablet, Roche). Cell lysis was carried out by sonication on ice (3-min in short 20-s pulses with 20-s intervals). Lysate was centrifuged at 12,000 g for 30 min and the pellet was discarded. The supernatant was loaded onto a HisTrap HP 5-ml column (GE Healthcare), previously equilibrated with buffer A. The column was washed with 50 mL of the same buffer, 60 ml of the same buffer containing 20 mM imidazole, and eluted with a linear 20–400 mM imidazole gradient (the buffer containing imidazole was adjusted to pH 8.0 with HCl). Collected fractions (1.0 mL) were analyzed by SDS-PAGE and those containing the expected protein were pooled and dialyzed overnight against 2 L of buffer A. When stated, 0.1 M sodium citrate was added to buffer A. Protein subunit concentration was calculated using a theoretical extinction coefficient at 280 nm of 27,390 and 30,370 M^−1^ cm^−1^ (calculated with the Expasy ProtParam tool) for *Ab*HemL and *Ab*HemA, respectively.

### Activity Assays

HemL activity was assayed by incubating a solution containing 0.7 μM *Ab*HemL in tricine buffer, pH 7.9, with 100 μM GSA at 37°C and stopping the reaction at different times by adding HClO_4_. The ALA formed was quantified with Ehrlich's reagent as described in Pugh et al. (1991). Total protein content was determined according to Bradford (1976).

### Size Exclusion Chromatography

Gel filtration of *Ab*HemA/L was performed on a Superdex 200 10/300 GL column (GE Healthcare, Little Chalfont, UK) at room temperature and at a flow rate of 0.5 mL/min in 50 mM Na phosphate buffer pH 8.0, containing 100 mM NaCl. In some cases, 0.1 M sodium citrate was added to the buffer. Elution profiles were obtained from absorbance at 280 nm. Calibration was performed with ferritin (440 kDa), aldolase (158 kDa) conalbumin (75 kDa), and ovalbumin (44 kDa).

### Gel Electrophoresis

SDS-PAGE was carried out according to Laemli. Native (not denaturing) gel was performed with 12.5% Acrylamide: N,N′-Methylene bis acrylamide 29:1 in TBE buffer.

## Results

### Cloning, Expression and Purification of AbHemA and AbHemL

The coding region of *AbhemA* (*HMPREF0010_01335*) and *AbhemL* (*HMPREF0010_02239*) from *A. baumannii* ATCC 19606 were cloned and expressed as N-terminal His-tagged proteins following the procedures detailed in Materials and Methods.

**Figure 1:**
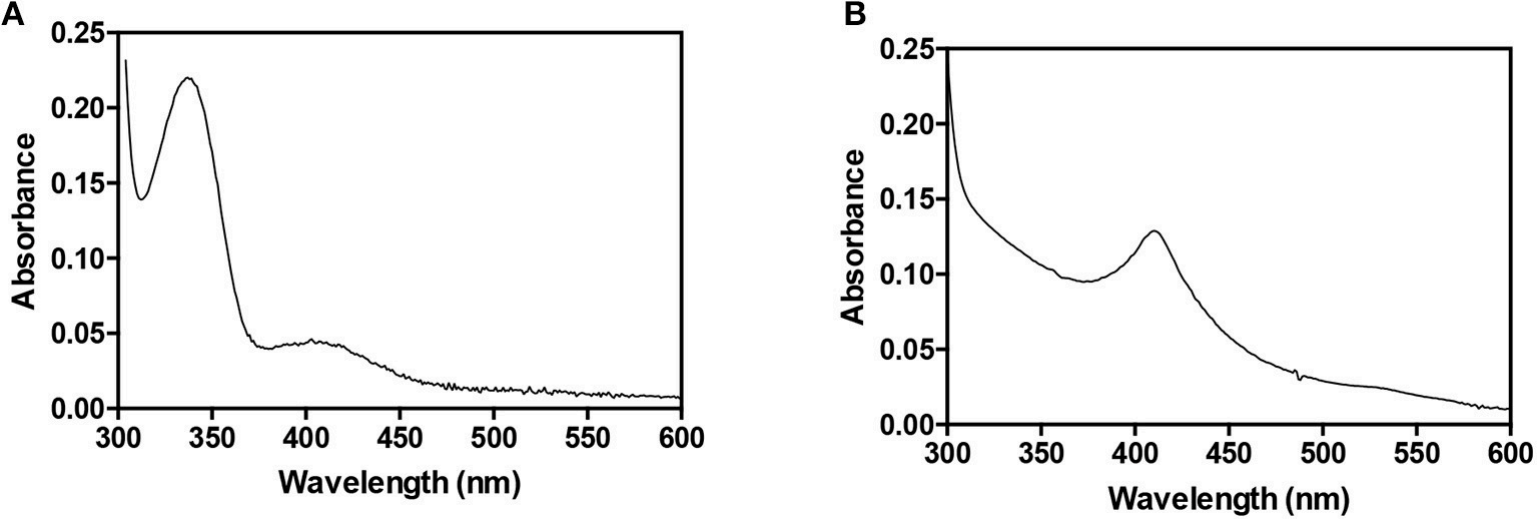
Absorption spectra of AbHemL and AbHemA. Spectra of 140 μM *Ab*HemL **(A)** and 34 μM *Ab*HemA **(B)** were recorded in 50 mM Na phosphate buffer, containing 0.3 M NaCl at 20°C using a Hewlett-Packard 8453 diode-array spectrophotometer.

*Ab*HemL was easily expressed and purified to homogeneity with a yield of 40 mg/l culture from *E. coli* BL21(DE3) strain containing pET28*hemL*_6xHis_. The UV-Vis absorption spectrum of purified HemL shows a major band at around 330 nm and a minor band at 410 nm (Figure 1A). These absorption bands are analogous to those observed with HemL from different sources (Berry-Lowe et al., 1992; Grimm et al., 1992; Pugh et al., 1992) and correspond, respectively, to PMP and PLP, as demonstrated from spectrophotometric measurements of the protein in NaOH (Peterson and Sober, 1954), which allowed their identification and quantification. In *A. baumannii* HemL, PLP is about 7% of the total protein-bound cofactor, and is expected to be bound as a Schiff base to an active site lysine residue (Lys270 in *Ab*HemL, as indicated by multiple sequence alignment; not shown).

On the contrary, our initial expression experiments of His-tagged *Ab*HemA in *E. coli* BL21(DE3) strain containing pET28*hemA*_6xHis_ gave poor results, as judged by SDS-PAGE analysis, and purification attempts gave low yield of a very unstable protein. Fruitlessly, we tried to improve the expression and purification yield, using different *E. coli* strains, such as HMS174(DE3) and Rosetta(DE3), and expression conditions, varying IPTG concentrations and growth temperatures. It is known that HemA, when complexed with heme, is the target of Lon and ClpAP proteases (Wang et al., 1999; Jones and Elliott, 2010; de Armas-Ricard et al., 2011). Because it has been reported that heme limitation leads to stabilization of HemA and therefore to an increase in enzyme abundance (Wang et al., 1999), ortho-phenanthroline, a cell-permeative iron chelator, was added to the culture medium in order to reduce iron availability for heme biosynthesis. In these conditions we observed a decrease in growth rate, but not an increase in *Ab*HemA expression. A fusion protein NusA-HemA, obtained by cloning the *hemA* gene in pET43 (obtaining pET43*hemA*_NusA_), was also generated that could be purified in soluble form and good yield. However, once cleaved from NusA and isolated, HemA proved to be poorly soluble and unstable.

All these observations suggested that HemA from *A. baumannii* is very difficult to express and purify in recombinant form, probably because it is very unstable inside bacterial cells. A possible solution to this problem was the co-expression of HemA with a protein that interacts with it and makes it more stable. A good candidate for this purpose was undoubtedly HemL. For this reason, two expression plasmids containing both *hemA* and *hemL* genes were constructed (Scheme 1). The first plasmid (pET28*hemL*_6xhis_-*hemA*_6xhis_) allows the expression of both proteins with an N-terminal His-tag, whereas the second plasmid (pET28*hemL-hemA*_6xHis_) leads to the expression of *Ab*HemA with an N-terminal His-tag and *Ab*HemL with no tags. When this latter plasmid was used for the co-expression of HemA and HemL in *E. coli* BL21(DE3) cells, a NiNTA affinity chromatography column, after extensive wash in order to get rid of all contaminants not binding to the resin, allowed the purification of *Ab*HemA to homogeneity as a stable protein, with a yield of 0.5 mg/liter of culture. The absorption spectrum of purified *Ab*HemA is shown in Figure 1B. The presence of an absorption band with maximum at about 410 nm, reminiscent of the Soret peak, and of a broad band at around 540 nm suggest that heme is possibly bound to the protein, as previously observed by other authors (de Armas-Ricard et al., 2011).

**Figure 2:**
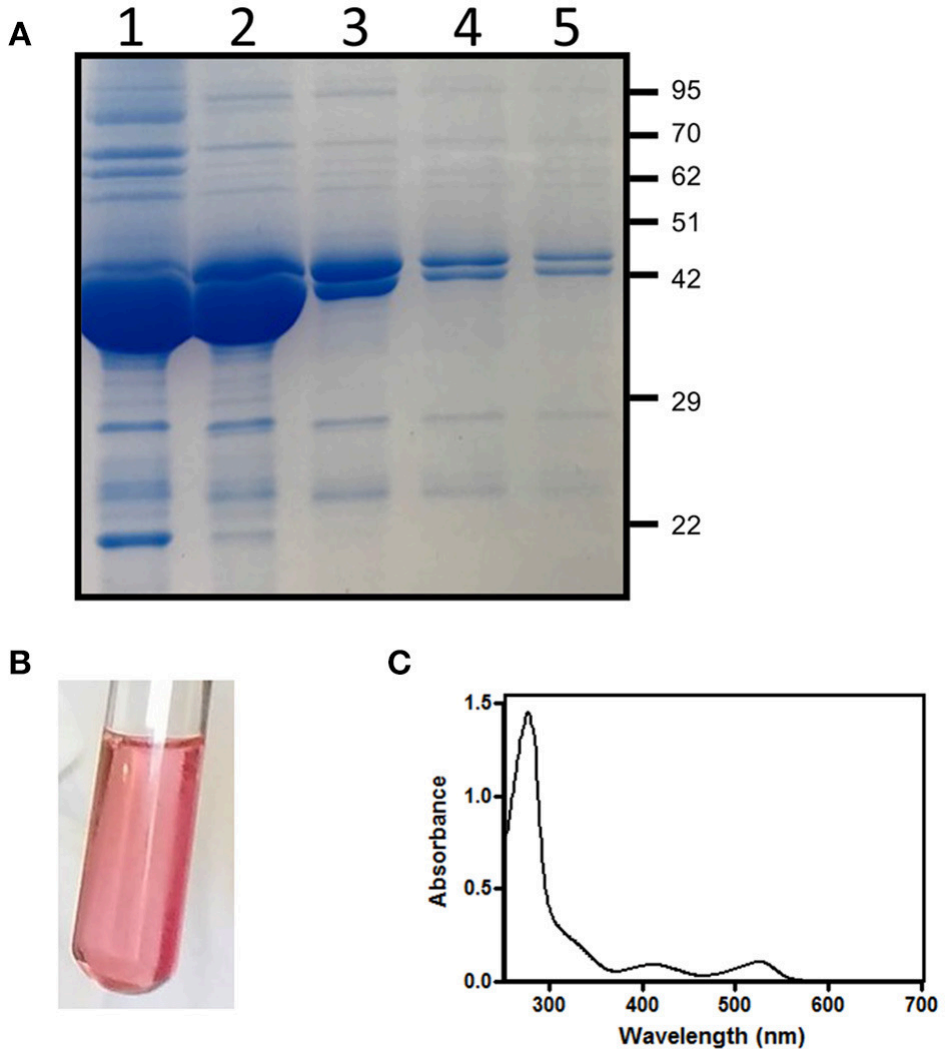
Purification of HemA-HemL complex. **(A)** SDS-PAGE analysis of fractions (from 1 to 5) from NiNTA chromatography column of *Ab*HemA-HemL complex purification. Aspect **(B)** and absorption spectrum **(C)** of fraction corresponding to lane 1.

*E. coli* cells containing the pET28*hemL*_6xHis_-*hemA*_6xHis_ vector allowed the co-purification of His-tagged *Ab*HemL (MW = 48.6 kDa) and *Ab*HemA (MW = 50.1 kDa) (Figure 2A). Fractions eluted from the NiNTA affinity chromatography column with about 160 mM imidazole were intense pink (Figure 2B) and mainly consisted of *Ab*HemL (Figure 2A lanes 1 and 2). Their absorption spectrum indicates the presence of a previously unobserved 520 nm absorbing species (Figure 2C), besides the PLP and PMP-dependent absorption bands characteristics of purified *Ab*HemL (see Figure 1A). Increasing the imidazole concentration, the eluate lost the pink color and the amount of *Ab*HemA with respect to *Ab*HemL also increased, as shown in the SDS-PAGE analysis (Figure 2A lanes 3–5).

### Effects of AbHemA and AbHemL Co-expression on Porphyrins and 5-Aminolevulinate Cellular Content

**Figure 3:**
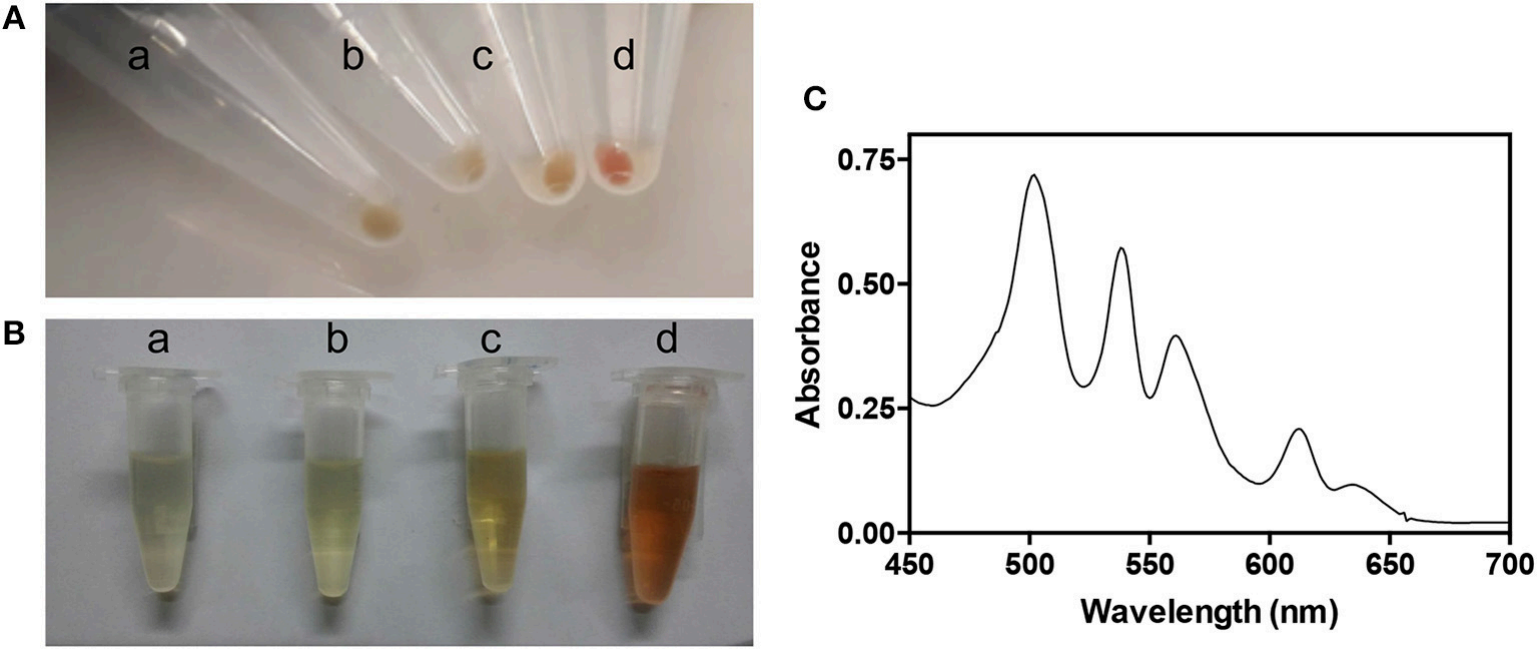
Characteristics of strains overexpressing AbHemL and AbHemA. Pellets **(A)** and lysates **(B)** from *E. coli* BL21(DE3) containing the empty vector pET28 (a), pET28*hemL*_6xHis_ (b), pET28*hemA*_6xHis_ (c), and pET28*hemL*_6xHis_-*hemA*_6xHis_ (d). **(C)** Absorption spectrum of lysate from *E. coli* BL21(DE3) containing pET28*hemL*_6xHis_-*hemA*_6xHis_.

During the purification procedure, we observed that the bacterial pellet and the lysate of *E. coli* BL21(DE3) cells co-expressing *Ab*HemA and *Ab*HemL (from strain containing both pET28*hemL*_6xHis_-*hemA*_6xHis_ and pET28*hemL-hemA*_6xHis_ plasmids) appeared reddish (Figures 3A,B). The visible region of the absorption spectrum of the bacterial lysate supernatant shows a series of peaks (Figure 3C) that are typical of porphyrins Q bands (Milgrom, 1997).

**Figure 4:**
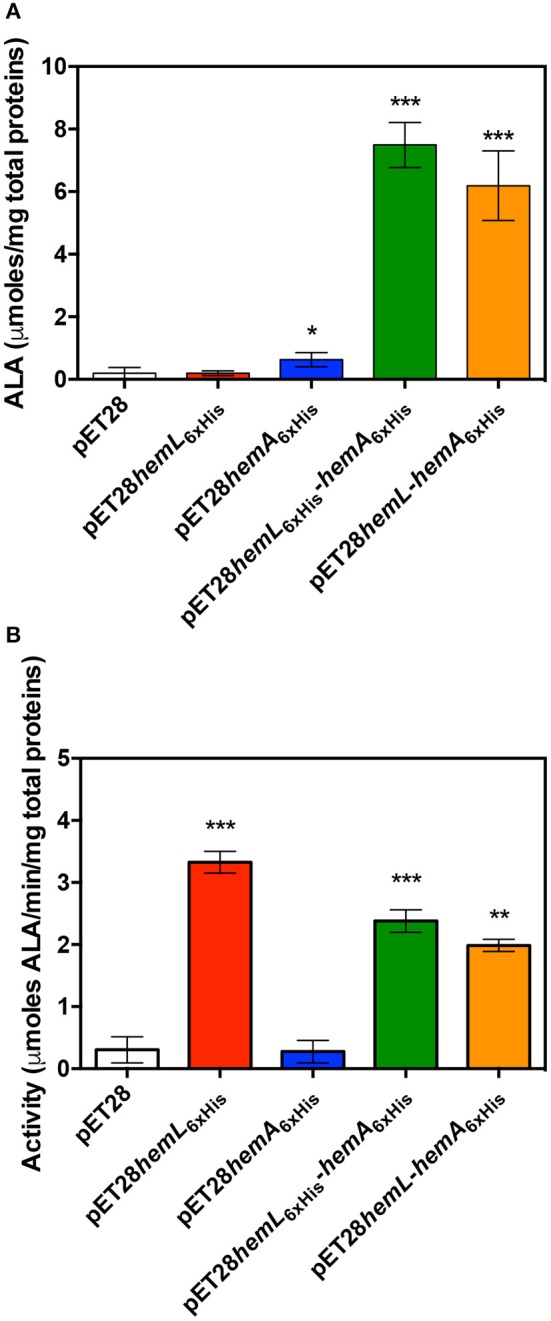
Effect of AbHemL and AbHemA overexpression. Content of ALA **(A)** and HemL activity **(B)** measured in lysate from *E. coli* BL21(DE3) containing the empty vector pET28, pET28*hemL*_6xHis_, pET28*hemA*_6xHis_, pET28*hemL*_6xHis_-*hemA*_6xHis_, and pET28*hemL*-*hemA*_6xHis_. Reported values are the mean ± standard deviation of four independent measurements. *P < 0.05, **P < 0.005, and ***P < 0.0005. Differences between HemL activity of strains containing pET28*hemL*_6xHis_-*hemA*_6xHis_ and pET28*hemL*-*hemA*_6xHis_ with respect to strain containing pET28*hemL*_6xHis_ are statistically significant (*P* < 0.05).

This high level of porphyrins content suggests that both overexpressed enzymes are active in the cells. For this reason, the content in ALA was determined in lysate supernatants of *E. coli* BL21(DE3) cells containing the empty pET28 vector, pET28*hemL*_6xHis_, pET28*hemA*_6xHis_, pET28*hemL*_6xHis_-*hemA*_6xHis_, and pET28*hemL-hemA*_6xHis_ (Figure 4A). When the co-expression of *Ab*HemA and *Ab*HemL was carried out with pET28*hemL*_6xHis_-*hemA*_6xHis_ and pET28*hemL-hemA*_6xHis_ plasmids, 7.5 ± 0.7 and 6.2 ± 0.5 μmoles of ALA per mg of total proteins were measured, respectively. When only *Ab*HemL was expressed (pET28*hemL*_6xHis_), no difference in the ALA content with respect to cells transformed with the empty vector was observed (0.2 ± 0.03 μmoles per mg of total proteins as compared to 0.2 ± 0.1 μmoles per mg of total proteins). Interestingly, cells containing pET28*hemA*_6xHis_, although not useful to express and purify *Ab*HemA, contained a slight, but statistically significant higher amount of ALA (0.6 ± 0.09 μmoles per mg of total proteins), with respect to the strain containing the empty vector. This result is not surprising, considering that formation of ALA might occur during the spontaneous degradation of GSA that takes place when this compound is not preserved at acidic pH (Hoober et al., 1988). This may be the reason why the knockout of the *hemL* gene results in bacteria that retain some growth capability even in the absence of ALA supplementation (Elliott et al., 1990; Hansson et al., 1991; Ilag et al., 1991). Therefore, the slightly higher amount of ALA measured in cells containing pET28*hemA*_6xHis_ may derive from GSA produced by HemA.

The enzyme activity of HemL was assayed in lysate supernatants from *E. coli* BL21(DE3) containing the empty pET28 vector, pET28*hemL*_6xHis_, pET28*hemA*_6xHis_, pET28*hemL*_6xHis_-*hemA*_6xHis_, and pET28*hemL-hemA*_6xHis_ (Figure 4B). In cells where *Ab*HemL only was overexpressed, a much higher activity, in terms of μmoles of ALA produced from GSA per minute per mg of total proteins, was measured with respect to strains containing either the empty pET28 or the pET28*hemA*_6xHis_ plasmids (3.3 ± 0.1 vs. 0.3 ± 0.1 and 0.27 ± 0.09). Remarkably, the co-expression of *Ab*HemA and AbHemL significantly dropped the HemL activity down. In fact, the activity was 2.4 ± 0.2 and 2.0 ± 0.07 in *E. coli* BL21(DE3) containing pET28*hemL*_6xHis_-*hemA*_6xHis_ and pET28*hemL-hemA*_6xHis_, respectively.

### When Co-expressed and Co-purified, AbHemA and AbHemL Form a Stable Complex

**Figure 5:**
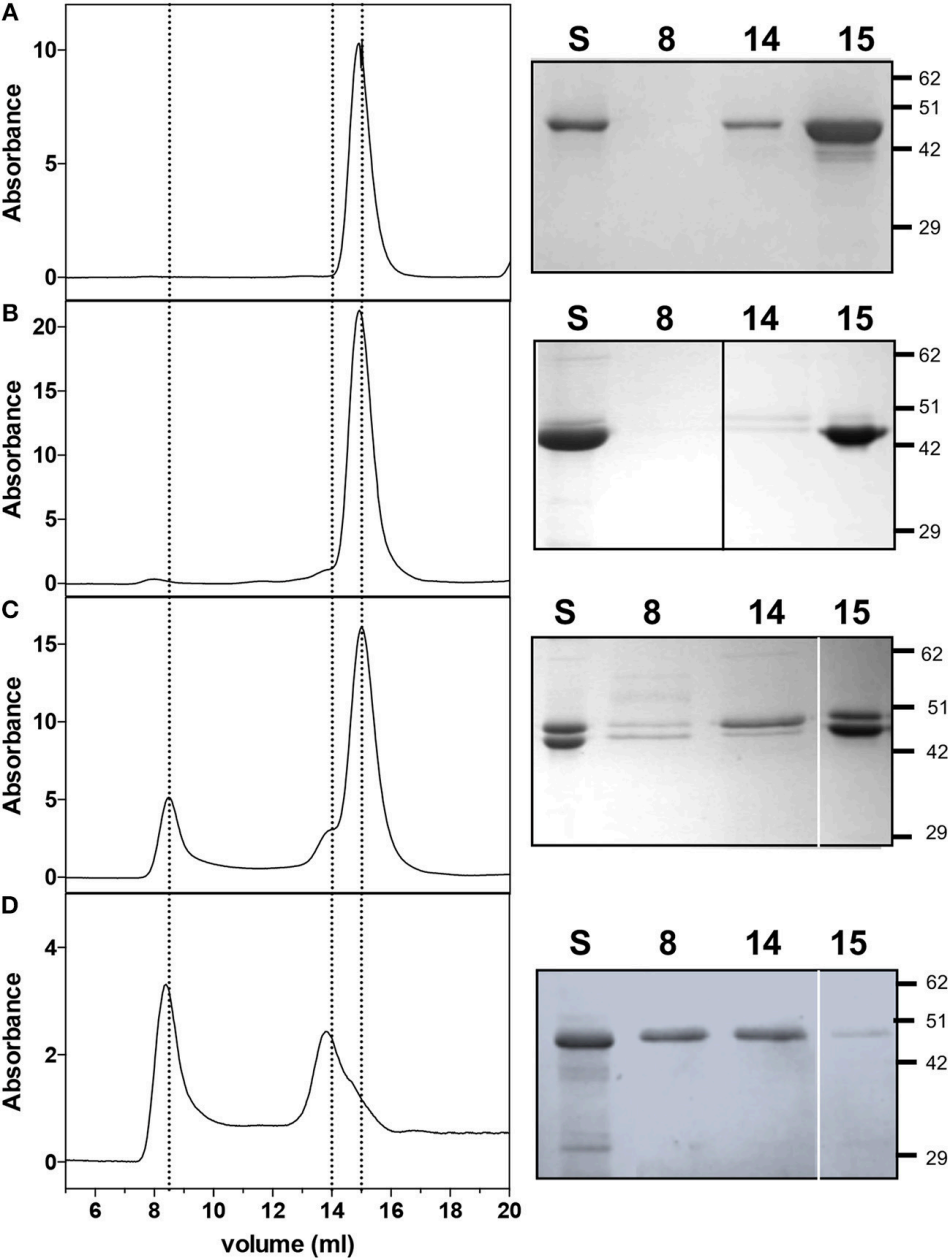
Size exclusion chromatography and SDS-PAGE analyses on AbHemL, HemA-HemL complex and AbHemA. Elution profiles of *Ab*HemL **(A)**, fractions from *Ab*HemA-HemL complex corresponding to lane 2 of Figure 2B and lane 3 from Figure 2C and *Ab*HemA **(D)**. Rigth panels are the SDS-PAGE of the same samples analyzed on SEC (S) and fractions from SEC at 8, 14, and 15 ml elution volume. In right panel **(B)**, the black line indicates that the image was assembled from two different gels. White lines on right panels **(C,D)** separate different parts of the same gel that were combined, excluding lanes which were not of interest. Original gels from which panels were assembled are shown in the Supplementary Materials.

The protein content of fractions obtained from the co-purification of HemA and HemL (from strain containing pET28*hemL*_6xHis_-*hemA*_6xHis_, see Figure 2A) was analyzed by size exclusion chromatography (SEC) and compared to individually purified *Ab*HemA and *Ab*HemL. *Ab*HemL purified from cells containing pET28*hemL*_6xHis_ gave a single peak at 14.9 ml elution volume (Figure 5A), which on the basis of the calibration curve corresponds to a molecular weight of about 89 kDa and therefore is consistent with a 97-kDa homodimer. The pink fractions containing a large amount of *Ab*HemL with respect to *Ab*HemA, such as in lane 1 and 2 of Figure 4A, gave a similar peak (Figure 5B left panel). The small amount of *Ab*HemA contained in these samples is represented in the shoulder at 14 ml (Figure 5B left panel). Fractions containing equal amounts of *Ab*HemA and *Ab*HemL (lanes 3 to 7 of Figure 4A) gave an additional peak at 8.5 ml elution volume (Figure 5C left panel), which started to be visible also in the previous sample. This peak corresponds to a molecular weight of about 200 kDa and, as the SDS-PAGE analysis revealed, contained both *Ab*HemA and *Ab*HemL (Figure 5C right panel). This result clearly indicates that *Ab*HemA and *Ab*HemL interact forming a stable complex that is maintained during the chromatographic separation. However, also individual *Ab*HemA and *Ab*HemL are present, as shown by the SEC and SDS-PAGE analyses (Figure 5C, 14 ml and 15 ml peaks). SEC and SDS-PAGE analyses of the individually *Ab*HemA, purified from *E. coli* strain containing pET28*hemL-hemA*_6xHis_, showed that this protein probably exists both as tetramer (8.5 ml elution volume) and as dimer (15 ml elution volume) (Figure 5D).

**Figure 6:**
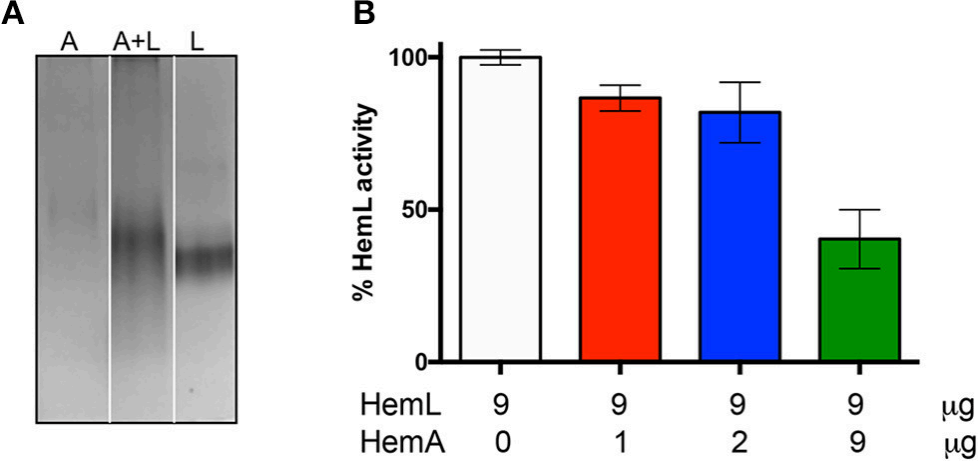
Effects of complex AbHemA-HemL formation. **(A)** Native gel on *Ab*HemA (A), *Ab*HemL (L) and the complex *Ab*HemA-HemL (A+L). White lines separate different parts of the same gel that were combined, excluding lanes which were not of interest. The original gel from which the panel was assembled is shown in the Supplementary Materials. It should be noticed that the amounts of *Ab*HemA and *Ab*HemL proteins analyzed in the gel were the same (2 μg each), as determined spectrophotometrically, although the protein band corresponding to *Ab*HemA is less intense than the band corresponding to *Ab*HemL. **(B)** HemL activity assays with the indicated amounts of *AbHemA* and *AbHemL*. Reported values are the mean ± standard deviation of four independent assays.

The formation of the *Ab*HemA-*Ab*HemL complex was also analyzed by native polyacrylamide gel electrophoresis. Figure 6A shows that migration of individually purified *Ab*HemA and *Ab*HemL, and co-purified *Ab*HemA and *Ab*HemL (lane 4 of Figure 4A) is different, indicating an interaction between the two proteins.

### HemL Catalytic Activity of the HemA-HemL Complex

On the basis of the results shown in Figure 4B, we hypothesized that when HemL interacts with HemA to form a complex, its activity may be decreased. In order to check this hypothesis, HemL activity was assayed in the different samples coming from *Ab*HemA and *Ab*HemL co-purification (see Figure 2A). Samples were prepared so that their *Ab*HemL concentration was identical (9 μg/μl), whereas *Ab*HemA varied from 1 to 9 μg/μl. The activity of these samples was compared to that of the individually purified *Ab*HemL (from strain containing pET28*hemL*_6xHis_), which was taken as 100% activity. Results in Figure 6B show that an increasing ratio of *Ab*HemA over *Ab*HemL affects HemL activity, which halves in samples where equal concentrations of *Ab*HemA and *Ab*HemL were present.

### Stabilization of the AbHemA-HemL Complex

**Figure 7:**
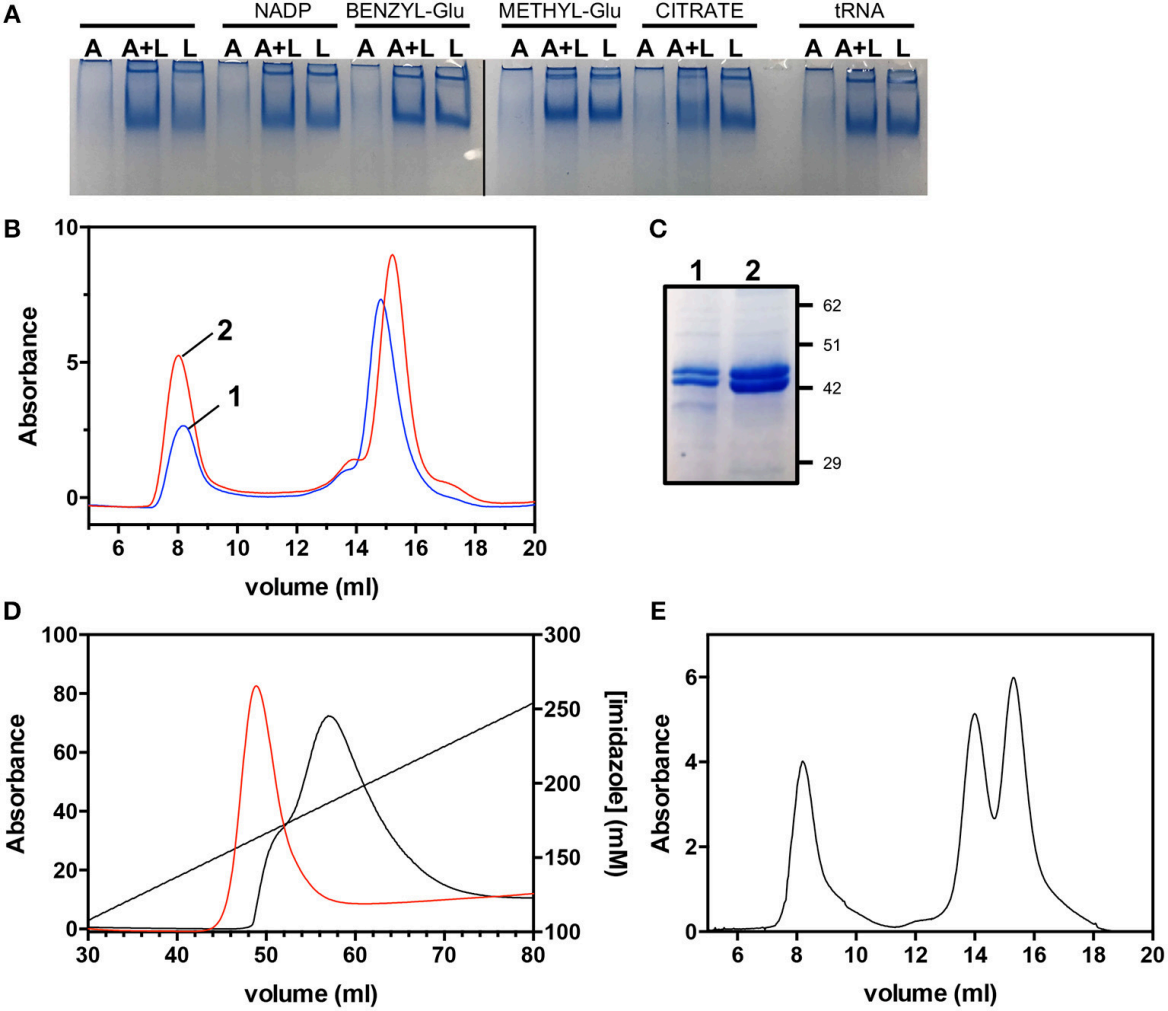
Effects of citrate on the AbHemA-HemL complex. **(A)** Native gel on *Ab*HemA (A), *Ab*HemL (L) and a mixture of same amounts (0.7 μg/μL) of *Ab*HemA and *Ab*HemL (A+L) in the presence of HemA ligands: 0.1 mM NADP^+^, 10 mM glutamate benzyl-ester (BENZYL-Glu), 10 mM glutamate methyl-ester (METHYL-Glu), 10 mM sodium citrate and 0.1 mM tRNA. The black line indicates that the image was assembled from two different gels. The original gels from which the panel was assembled are shown in the Supplementary Materials. **(B)** Size exclusion chromatography of *Ab*HemA-HemL complex in the presence (red line) or absence (black line) of 0.1 M sodium citrate. **(C)** SDS-PAGE analysis of fractions from SEC (sample 1 and 2) at 8 ml elution volume. **(D)** Affinity chromatography elution profiles obtained in different purification procedures of co-expressed *Ab*HemA and *Ab*HemL, carried out in the absence (black line) or the presence (red line) of 0.1 M sodium citrate. **(E)** Size exclusion chromatography of the *Ab*HemA-HemL sample purified in the presence of 0.1 M sodium citrate.

Mixtures of individually purified *Ab*HemL and *Ab*HemA, containing different concentration ratios of HemA and HemL (4:1, 2:1, 1:1, and 1:2), were analyzed by native polyacrylamide gel electrophoresis and SEC, but no evidence of complex formation was obtained. Enzymatic assays on the same mixtures showed that the *Ab*HemL activity was not affected by the presence of *Ab*HemA, even when this enzyme was present in a four-fold molar excess with respect to *Ab*HemL (data not shown). These results indicate that the *Ab*HemA-HemL complex isolated when the enzymes are co-expressed and co-purified is probably formed thanks to the particular conditions present inside the bacterial cells. In the attempt to mimic such conditions, we incubated 1:1 mixtures of the two individually purified proteins in the presence of different HemA ligands, such as tRNA, NADP^+^, citrate [which is required for the successful crystallization of HemA from *M. kandleri* (Moser et al., 2001)] and two substrate analogs, i.e., glutamate methyl-ester and glutamate benzyl-ester. These different mixtures were analyzed by native polyacrylamide gel electrophoresis. Figure 7A shows that the only hint of a HemA-HemL complex was obtained in the presence of 10 mM sodium citrate. In order to check if the presence of citrate stabilizes the *Ab*HemA-HemL complex, two identical protein samples (2.5 mg each) coming from the co-purification of *Ab*HemA and *Ab*HemL (Figure 2A) and containing equal amounts of the two proteins (Figure 5C) were incubated at 4°C for 16 h in the absence or presence of 0.1 M sodium citrate and analyzed by SEC. Figures 7B,C show that a net increase (about four-fold as estimated spectrophotometrically and from the intensity of Coomassie-stained bands in Figure 7C) of the 200 kDa-protein fraction eluted at 8 ml from the SEC column, corresponding to the *Ab*HemA-HemL complex, was observed when the *Ab*HemA-HemL mixture was incubated in the presence of citrate (peak 2 in Figure 7B). Analogously, the amount of *Ab*HemA-HemL complex that could be isolated when the proteins were co-expressed (using the pET28*hemL*_6xHis_-*hemA*_6xHis_ plasmid) increased when bacteria were grown in LB medium containing 20 mM sodium citrate, and 0.1 M sodium citrate was included in all buffers used in the purification procedure. The increased amount of complex is evident in the elution profile of the affinity chromatography (Figure 7D) and confirmed by the following SEC analysis (Figure 7E). Differently from what observed in the absence of citrate, *Ab*HemA and *Ab*HemL co-eluted from the affinity chromatography with 150-180 mM imidazole. Moreover, in the SEC analysis the integration of peak areas (see Figure 7E, compared to Figure 5C) demonstrated that the ratio between the *Ab*HemA-HemL complex and the single dimeric proteins was increased. It could be estimated that about 0.6 mg of *Ab*HemA-HemL complex per liter of bacterial culture can be isolated following this procedure.

## Discussion

Antimicrobial resistance is a problem of global dimension as frequently echoed by media, experts and public health agencies. *A. baumannii* is one of the most problematic microorganisms because of its ability to escape from most common antibacterial treatments (Antunes et al., 2014). Indeed this organism has been included in the priority 1 (critical) group in the list of antibiotic resistant pathogens published by the World Health Organization (http://www.who.int/news-room/detail/27-02-2017-who-publishes-list-of-bacteria-for-which-new-antibiotics-are-urgently-needed). The two-step pathway catalyzed by HemA and HemL is considered to be the rate-limiting step in the biosynthesis of tetrapyrroles, which is unique to plants and bacteria. Therefore, HemA and HemL represent very promising targets for the development of novel antibiotic treatments of *A. baumannii*, as well as potential herbicides. Some inhibitors of HemL have been tested *in vitro* (Grimm et al., 1991; Tyacke et al., 1995; Contestabile et al., 2000b), however, none of these compounds possessed the specificity and efficiency required to act as antibacterial agents. Moreover, three promising inhibitors of HemA were also identified (Loida et al., 1999), although they were never developed as antibiotics. Since it is known that HemA and HemL carry out their biological function in the form of a complex (Moser et al., 2001; Lüer et al., 2005; Nogaj and Beale, 2005), this feature should be considered when a drug design approach has to be applied to the development of inhibitors of these enzymes. In fact, the active site structure of HemA and HemL in the complex may substantially differ from that of the individual enzymes. Moreover, the mechanism of the catalyzed reactions based on channeling may be far from the mechanism of the single enzymes. In this contest, it is of fundamental importance to obtain sufficient amounts of stable HemA-HemL complex in order to characterize its functional properties and solve its three-dimensional structure. Although other authors have already demonstrated the existence of this complex, its isolation has never been attained. The instability of HemA surely represented a bottleneck in the achievement of this task. This protein is protease-sensitive and is strictly regulated by heme cellular concentration (Wang et al., 1999; Jones and Elliott, 2010; de Armas-Ricard et al., 2011). We have been able to purify a fair amount of *Ab*HemA (0.5 mg/L of culture) only when this protein was co-expressed in an *E. coli* strain containing the pET28*hemL-hemA*_6xHis_ plasmid, which led to production of His-tagged *Ab*HemA and untagged *Ab*HemL (Scheme 1). This is probably because the overexpression of *Ab*HemL protects *Ab*HemA from degradation. Co-expression of these proteins had already been devised, although with the precise purpose to produce ALA by bacterial fermentation (Kang et al., 2011; Zhang et al., 2015). In our case, when *Ab*HemL and *Ab*HemA were co-expressed, more than 6 μmoles of ALA per mg of total proteins were produced (Figure 4A) and evidence of porphyrins production was obtained (Figure 3). However, the co-expression strategy was applied in this work to isolate a significant amount of complex (Figures 5C, 7E) suitable for future crystallographic studies. It is worth noticing that the catalytic activity of *Ab*HemL (the conversion of GSA into ALA) decreases when this enzyme is complexed with *Ab*HemA (Figures 4B, 6B). Such a decrease, which we take as proof of the formation of a complex between *Ab*HemA and *Ab*HemL, could derive from a reduced accessibility of the active site of *Ab*HemL. In fact, in the model of the complex resulting from the docking of *M. kandleri* HemA and *Synechococcus* HemL, the active site entrance of the latter enzyme is located right in front of a depression of the HemA catalytic domain, suggesting that the GSA produced in the HemA active site may be directly channeled to HemL, without exposure to the aqueous environment (Moser et al., 2001). In our activity assay, GSA, which is not produced by HemA but is supplied externally, may encounter difficulties to reach the HemL active site.

The addition of citrate to the growth medium and to the buffers used in the purification procedure increased the amount of *Ab*HemA-HemL complex that could be isolated. This observation is not surprising, considering that citrate proved to be mandatory for the crystallization of *M. kandleri* HemA, presumably by stabilizing an open, “pre-active” conformation of the enzyme (Moser et al., 2001). Notably, Arg300, which in the crystal structure of *M. kandleri* HemA binds the citrate anion with a total of three different hydrogen bonds, is conserved in *Ab*HemA. It has been hypothesized that the citrate anion may partly mimic the acceptor stem of the glutamyl-tRNAGlu substrate of HemA, either as a counter ion for the backbone phosphate or for specific base recognition (Moser et al., 2001). It is therefore plausible that binding of citrate to *Ab*HemA promotes the formation of the *Ab*HemA-HemL complex.

## Conclusions

This work presents a useful method that allows the purification of the complex formed by HemA and HemL from *A. baumannii* and lays the basis for its future characterization. These are pivotal enzymes in the biosynthesis of ALA, the universal precursor of tetrapyrroles such as heme and chlorophylls, and represent relevant biomedical targets. In fact, a detailed knowledge of the structural and functional properties of the complex represents the starting point for the rational design of specific and efficient inhibitors that may be developed as antibacterial agents.

## Author Contributions

CN, DB, and AB performed the experiments. MdS and JS critically read, analyzed and discussed the literature, and the results of the experiments. AT performed and designed the experiments. AT and RC wrote the manuscript and supervised the project. All the authors edited the manuscript and provided valuable discussions and criticisms.

### Conflict of Interest Statement

The authors declare that the research was conducted in the absence of any commercial or financial relationships that could be construed as a potential conflict of interest.

## Abbreviations

ALA: 5-Aminolevulinic acid
GSA: glutamate-1-semialdehyde
PLP: pyridoxal 5′-phosphate
PMP: pyridoxamine 5′-phosphate
DAVA, 4: 5-diaminovalerate
*Ab*HemA: *Acinetobacter baumannii* HemA
*Ab*HemL: *Acinetobacter baumannii* HemL
SEC: size exclusion chromatography.
