# Supplementary material for: Isolation of a Complex Formed Between Acinetobacter baumannii HemA and HemL, Key Enzymes of Tetrapyrroles Biosynthesis
Source: Front Mol Biosci. 2019 Feb 26;6:6. doi: 10.3389/fmolb.2019.00006 (PMC6399207; doi:10.3389/fmolb.2019.00006)

Figure 5
Left panels are those present in the submitted manuscript. On the right, the original gels (SDS-PAGE) used to assemble the panels are shown. The first gel contains: the molecular weight standard (Std), sample 14 of panel B (B14) and sample 15 of panel B (B15). The second gel contains: sample S of panel B (BS), sample 8 of panel B (B8), sample S of panel C (CS), sample 8 of panel C (C8), sample 14 of panel C (C14), an intermediate sample which was not shown in the figure (14.5), sample 15 of panel C (C15) and, after an empty lane, the molecular weight standard (Std). The third gel contains: sample 15 of panel D (D15), the molecular weight standard (Std), sample S of panel D (DS), an empty lane, sample 8 of panel D (D8) and sample 14 of panel D (D14).

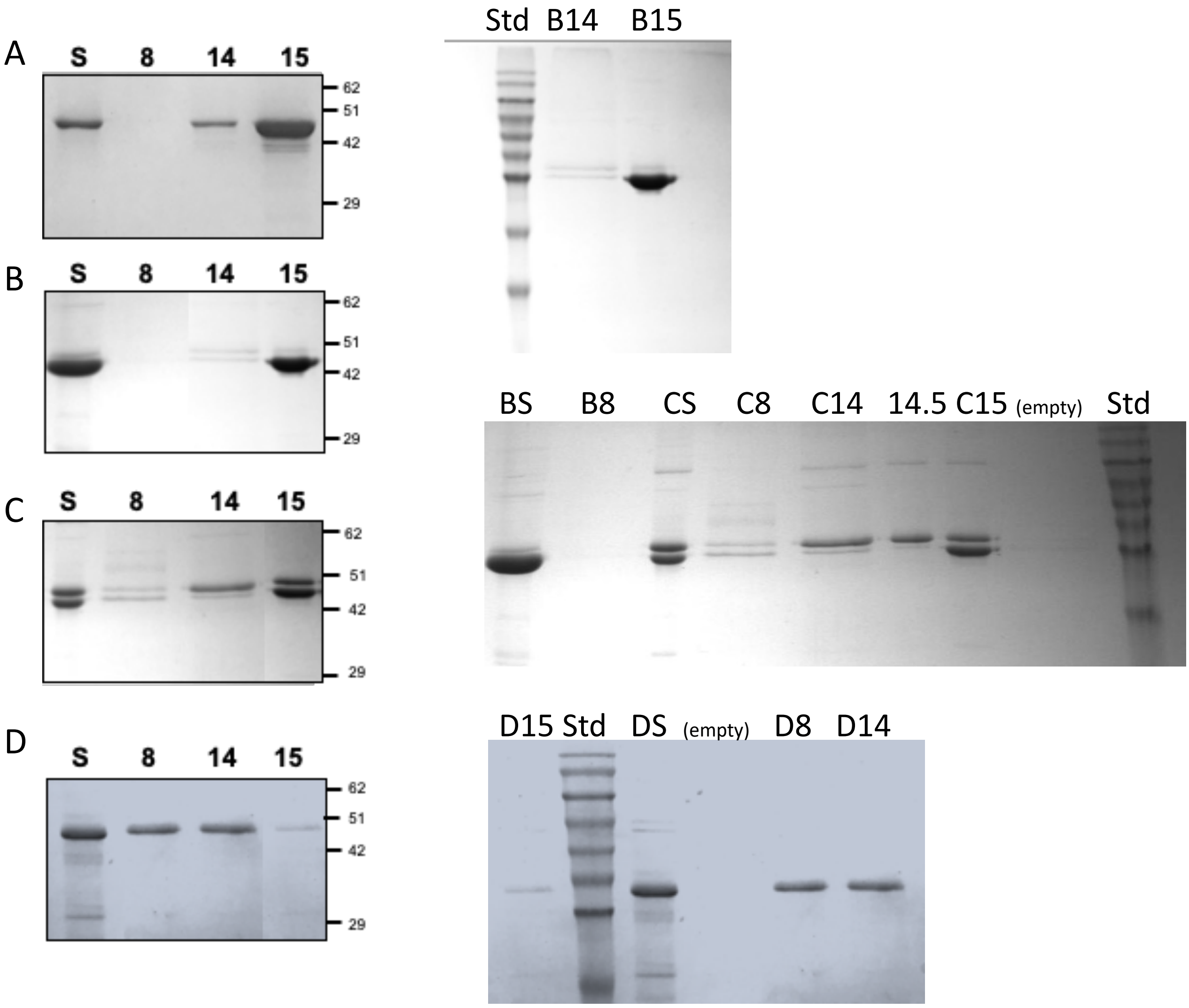

Figure 6

Panel A of figure 6 (reported on the left) was made using three lanes of the native gel shown on the right, as indicated by the arrows.

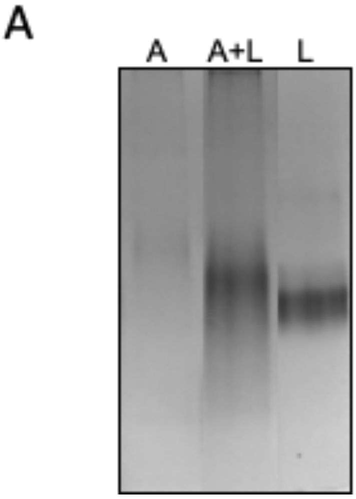

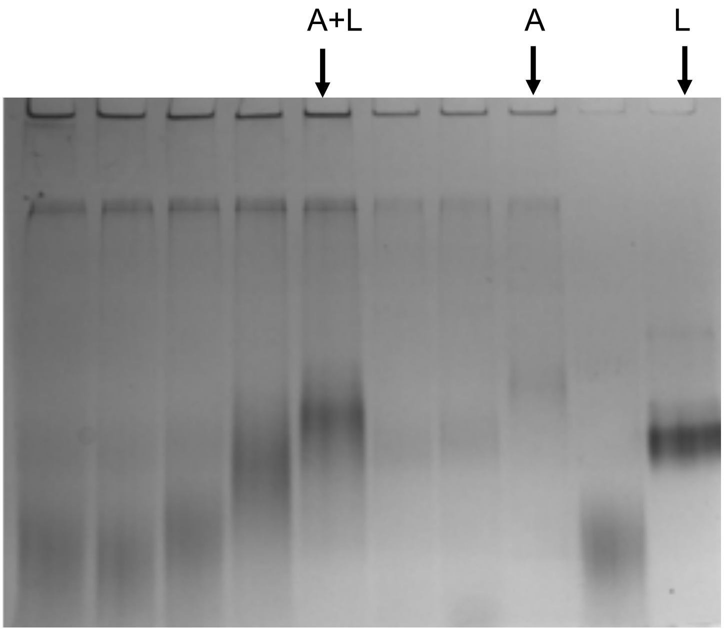

Figure 7

The panel A of figure 7 was made by the two native gels reported below.

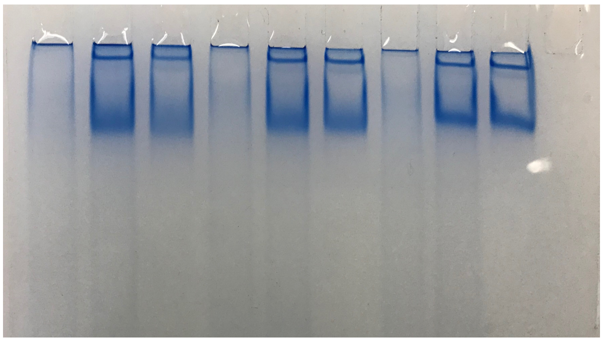

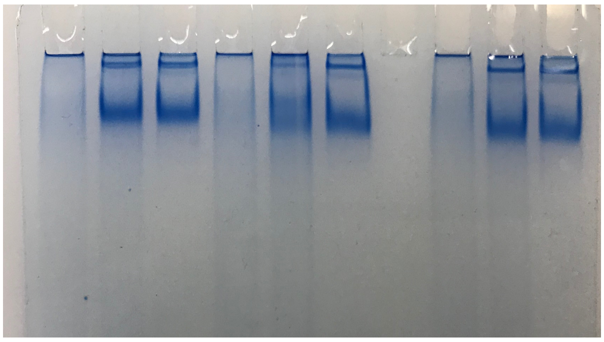

Supplement: Supplementary file 1 [file Data_Sheet_1.PDF]
